# *Brosimum alicastrum* Sw. (Ramón): An Alternative to Improve the Nutritional Properties and Functional Potential of the Wheat Flour Tortilla

**DOI:** 10.3390/foods8120613

**Published:** 2019-11-24

**Authors:** Rodrigo Subiria-Cueto, Alfonso Larqué-Saavedra, María L. Reyes-Vega, Laura A. de la Rosa, Laura E. Santana-Contreras, Marcela Gaytán-Martínez, Alma A. Vázquez-Flores, Joaquín Rodrigo-García, Alba Y. Corral-Avitia, José A. Núñez-Gastélum, Nina R. Martínez-Ruiz

**Affiliations:** 1 Instituto de Ciencias Biomédicas, Universidad Autónoma de Ciudad Juárez, Anillo Envolvente del Pronaf y Estocolmo s/n, C.P. Ciudad Juárez, Chihuahua 32310, Mexico; al183351@alumnos.uacj.mx (R.S.-C.); ldelaros@uacj.mx (L.A.d.l.R.); lsantana@uacj.mx (L.E.S.-C.); alma.vazquez@uacj.mx (A.A.V.-F.); jogarcia@uacj.mx (J.R.-G.); acorral@uacj.mx (A.Y.C.-A.); jose.nunez@uacj.mx (J.A.N.-G.); 2 Unidad de Recursos Naturales, Centro de Investigación Científica de Yucatán, A.C. (CICY), Calle 43, No. 130 x 32 y 34, Chuburná de Hidalgo, C.P. Mérida, Yucatán 97205, Mexico; larque@cicy.mx; 3 Programa de Posgrado en Alimentos, Universidad Autónoma de Querétaro, Cerro de las Campanas s/n, Col. Las Campanas, Santiago de Querétaro, Querétaro 76100, Mexico; luzrega@icloud.com (M.L.R.-V.); marcelagaytanm@yahoo.com.mx (M.G.-M.)

**Keywords:** nutritional value, antioxidant capacity, phenolic compounds, sensory properties, functional foods

## Abstract

The wheat flour tortilla (WFT) is a Mexican food product widely consumed in the world, despite lacking fiber and micronutrients. Ramón seed flour (RSF) is an underutilized natural resource rich in fiber, minerals and bioactive compounds that can be used to improve properties of starchy foods, such as WFT. The study evaluated the impact of partial replacement of wheat flour with RSF on the physicochemical, sensory, rheological and nutritional properties and antioxidant capacity (AC) of RSF-containing flour tortilla (RFT). Results indicated that RFT (25% RSF) had higher dietary fiber (4.5 times) and mineral (8.8%; potassium 42.8%, copper 33%) content than WFT. Two sensory attributes were significantly different between RTF and WFT, color intensity and rollability. RFT was soft and it was accepted by the consumer. Phenolic compounds (PC) and AC were higher in RFT (11.7 times, 33%–50%, respectively) than WFT. PC identification by ultra-performance liquid chromatography quadrupole time of flight mass spectrometry (UPLC-QTOF-MS) showed that phenolic acids esterified with quinic acid, such as chlorogenic and other caffeoyl and coumaroyl derivatives were the major PC identified in RSF, resveratrol was also detected. These results show that RSF can be used as an ingredient to improve nutritional and antioxidant properties of traditional foods, such as the WFT.

## 1. Introduction

Tortilla is an iconic food in the Mexican and Central American diet. Mexico is the main tortilla consumer around the world (75 kg/person) [1], with 11.5 million tons consumption per year [2]. The growing demand of tortilla has caused its globalization, representing nowadays a food of importance in countries such as USA or China. Tortilla is usually made from wheat or corn [3]. On the one hand, the corn tortilla provides proteins, calcium, potassium and carbohydrates, which makes this food an important vehicle to provide nutrients to people with malnutrition. On the other hand, the wheat flour tortilla (WFT) provides more calories (47%), proteins, lipids and carbohydrates and less dietary fiber and micronutrients than corn tortilla [4], which makes this type of tortilla a hypercaloric and a poorly nutritious food. However, WFT has a wide demand in northern Mexico and USA and it is highly consumed by children, youth and adults from ethnic and low-income populations contributing to exacerbate states of undernourishment [5] or obesity [6]. Malnutrition is one of the main public health problems affecting many countries [7] and is associated, in many cases, with the high intake of hypercaloric foods with low dietary fiber, minerals and vitamins [6]. Therefore, it is of interest to make culturally rooted foods, such as tortilla, with greater nutritional value in order to contribute to a healthier diet of the population; particularly for groups in poverty and/or malnutrition.

*Brosimum alicastrum* Sw. (ramón) is a tree of the Mexican tropics whose fruit and seed have high nutritional value. This tree was appreciated by the Mayan culture from 300 to 900 A.D. [8]. The flour obtained from the seed (RSF) is characterized by high protein, dietary fiber and micronutrient content. *Brosimum alicastrum* Sw. is considered as an underexploited natural resource in Mexico with potential nutritional and functional properties [9], which can be incorporated into foods with little nutritional value. In this context, the aim of this study was to evaluate the impact of partial replacement of wheat flour with RSF on the physicochemical, sensory, rheological and nutritional properties and antioxidant capacity of RSF-containing flour tortilla (RFT). The hypothesis proposed was that the partial replacement of WF with RSF in the tortilla improves its nutritional contribution in dietary fiber and micronutrient content, increases its antioxidant capacity and the RFT is sensory accepted by the consumer compared as well as a tortilla made with 100% WF.

## 2. Materials and Methods

### 2.1. Materials

The ramón seed flour (RSF) (*B. alicastrum* Sw.) was provided by CICY (Herbarium Roger Orellana, Centro de Investigación Científica de Yucatán A.C.). The seeds were collected from growing ramón trees at rancho Xoccheila (20°33′ N; 89°34′ W), municipality of Sacalum, Yucatán. The seeds were dried in the sun, the testa was removed and the seed was ground to a fine flour. The wheat flour (WF), dry (salt, baking powder) and moist (shortening) ingredients were purchased in the local markets of Ciudad Juárez, Chihuahua, México.

### 2.2. Tortilla Development

Different formulations were proposed partially replacing the content of WF for RSF (20%, 25%, 28%, 30% and 40%). The ramón flour tortilla (RFT) and the wheat flour tortilla (WFT) were developed following the previously reported method with some modifications [10]. Extra water was added to the RSF doughs until a more elastic texture was obtained. Microbiological quality in total coliforms bacteria (CC), aerobics mesophilic bacteria (AC), yeast and mold (YM) was determined in tortillas samples according to the plate count method (3M™, Petrifilm™, Minneapolis, MN, USA). Briefly, tortilla samples were diluted 1:10 in saline solution (0.9%), homogenized and one mL plated onto Petrifilm. Plates were incubated at 35 ± 1 °C for 24 h for CC, 48 h for AC and 25 ± 1 °C for YM. Official Mexican regulation was observed for the limits of CC, AC and YM in tortillas [11].

### 2.3. Proximate Composition, pH, Activity Water (Aw) and Titratable Acidity

All tortilla samples were homogenized using a commercial blender (Nutribullet^®^). The samples were analyzed in triplicate following the AOAC methods [12]: ash was determined in muffle furnace (Felisa^®^, Model FE-340, Jalisco, México) at 550 °C for 5 h; crude protein by Kjeldahl method (Labconco^®^, Model RapidStill II, Kansas city, MO, USA); fat by Soxhlet method (Soxtec™, Model 2043, Foss™, Hilleroed, Denmark); total carbohydrates by difference method; crude fiber by gravimetric method, dietary fiber by enzymatic-gravimetric assay, water activity in AQUA LAB^®^ (Model Serie 3, Meter Food, Washington, D.C., USA) equipment; pH and titratable acidity by potentiometric method (Accumet^®^, Model AB15 Plus, Westford, MA, USA). Moisture analysis was performed by the AOAC method [12] with the following modifications: it was determined in an oven (VWR^®^, Model 1324, Irving, TX, USA) at 105 °C for 8 h.

### 2.4. Extraction and Quantification of Minerals

The mineral content (Cu, Zn, K, Fe and Na) was obtained from the ashes of the samples following the method by the AOAC with minor modifications. Flours and tortilla samples (5 g) were calcined in a muffle furnace (Felisa^®^, Model FE-340, Guadalajara, Jalisco, México) for 8 h at 550 °C. Subsequently, 3 mL of HNO_3_ (SCP^®^, Quebec, Canada) (0.2%, *v*/*v*) was added and taken to dryness on a hot plate (100 °C). The samples were calcined again for 2 h at 550 °C until white ashes were obtained, 5 mL of 6 M HCl (JT Baker^®^, Fisher Scientific, West Palm Beach, Florida, USA) were added and dried. Finally, 20 mL of HNO_3_ (0.2%, *v*/*v*) (Merck^®^, Toluca, Estado de México, Mexico) were added and samples were transferred into plastic conical tubes (Corning^®^, Merck, Toluca, Estado de México, México). The samples were analyzed using atomic absorption spectroscopy (Perkin Elmer^®^, Model AAnalyst 200, Madrid, Spain) with acetylene flame adjusting the wavelength for each mineral [12].

### 2.5. Extraction and Quantification of Carotenoids

The content of carotenoids was determined following the method by Moreno-Escamilla et al. [13] with some modifications. Flours and tortillas were dried at 45 °C in a vacuum oven (Shel Lab^®^, Model VWR A-143, Tualatin, OR, USA) at 20 mm Hg for 36 h. Next, they were ground using a commercial blender (Nutribullet^®^) and kept at −18 °C in darkness for no more than 48 h. Subsequently, 0.3 g of dry ground sample were mixed with 10 mL of acetone (JT Baker^®^, Fisher Scientific, West Palm Beach, FL, USA), sonicated (Branson^®^, Model 5800, Fisher Scientific, West Palm Beach, Florida, USA) for 20 min and centrifuged (Eppendorf^®^, Model 5810 R, Alto da lapa, Sâo Paulo, Brazil) at 3500 rpm for 10 min. The supernatant was recovered, and the residue was extracted two more times under the same conditions. The absorbance of the combined supernatants was read in an ultraviolet–visible (UV–Vis) microplate reader (BioRad^®^, Model XMark, Ciudad de México, México) at a wavelength of 474 nm. Results were reported as milligrams of β-carotenoids per 100 g of sample.

### 2.6. Extraction and Quantification of Ascorbic Acid

Ascorbic acid determination was realized according to the technique described by Alvarez-Parrilla et al. [14], with some modifications. Extracts were obtained by mixing 0.2 g of flour or tortilla samples (DW) with 5 mL of 5% metaphosphoric acid (Merck^®^, Toluca, Estado de México, México), the mixture was sonicated (Branson^®^, Model 5800 Fisher Scientific, West Palm Beach, FL, USA) for 20 min in dark conditions, and centrifuged (Eppendorf^®^, Model 5810 R, Alto da lapa, Sâo Paulo, Brazil) at 3500 rpm for 10 min. For quantification, 300 μL of each supernatant was taken and mixed with 200 μL of 6.65% tricloriacetic acid (Merck^®^, Toluca, Estado de México, México) and 75 μL of the DNPH (2,4-dinitrophenylhydrazine) reagent (Merck^®^, Toluca, Estado de México, México) in 100 mL of 5 M H_2_SO_4_ (JT Baker^®^, West Palm Beach, Fisher Scientific, FL, USA), then incubated for 3 h at 37 °C and 0.5 mL of H_2_SO_4_ (JT Baker^®^, Fisher Scientific, West Palm Beach, FL, USA) (65%, *v*/*v*). Absorbance was measured in the UV-Vis microplate reader (BioRad^®^, Model xMark, Ciudad de México, México) at 520 nm, using ascorbic acid as standard. Results were reported as milligrams of ascorbic acid per 100 g of sample.

### 2.7. Sensory Characterization

Tortillas (RFT and WFT) were sensory characterized with a descriptive analysis by a trained panel of 8 judges. The attributes in the olfactory phase were odor intensity and descriptors determined by focus group technique. In the oral phase, mouthfeel characteristics were evaluated: such as cohesiveness, hardness, moistness, adhesiveness and astringency; taste: such as sour, salty and bitter. Color and texture attributes, such as elasticity, firmness and rollability were also evaluated. All tests were conducted in individual booths and the judges used a 150 mm linear scale, labeled at the end as “Not all…” and “Extremely …” for each attribute or descriptor. Each judge was provided with slices of tortilla (10 g), previously heated in a microwave for 30 s, and they were placed in 2 oz plastic cups, identified with three-digit random numbers. The samples were served to the judges in a balanced and randomized form, together with evaluation sheets. Judges rinsed their mouths with purified water (Alaska^®^, Chihuahua, Mexico) at the beginning and between samples for the oral phase and they used eye covers in all tests, except in the visual phase. Pantone^®^ scale was used in color test. Two attributes or descriptors were evaluated per session of 60 min, standards for each attribute or descriptor were used at the beginning of the test and each test was performed by duplicate [15,16].

### 2.8. Consumer Acceptance Test

An acceptance test was carried out to evaluate the consumer degree of liking for RFT and WFT. The test was performed in 120 consumers using a 9-point hedonic scale, ranging from “Like extremely” to “Dislike extremely”. Participants were given 5 g of RFT or WFT (freshly made) and kept warm in thermal containers (35 ± 2 °C) for a limited time of 15 min. Two samples were evaluated by session and each sample was presented monadically in disposable dishes (12 cm diameter), labeled with three-digit random numbers. Participants rinsed their mouths with purified water (Alaska^®^, Chihuahua, Mexico) at the beginning of the session and between samples. They tasted each sample and indicated on the hedonic scale the degree of liking for the sample [16].

### 2.9. Rheological Measurements

For the rheological characterization, texture profile analysis (TPA) and cohesiveness tests were performed in the RFT and WFT dough samples using the methods described by Reyes-Vega et al. [17] and Flores-Farías et al. [18] with modifications. Spherical dough fractions (2.0 cm diameter) were tested to obtain gumminess (N), hardness (N), adhesiveness (J), elasticity (mm) and chewiness (Nm). Cohesiveness was performed in dough samples (20 g) placed in a cylindrical container and a penetration speed of 2.0 cm·s^−1^ was applied. In the tortilla samples (2 × 6 cm), cutting, elongation and extensibility tests were made to obtain the force (N) required to separate it, the elongation (mm), the distance that can be stretched before breaking (mm), work necessary for extension (J) and cohesiveness (N) following the methods by Kelekei et al. [19] and Suhendro et al. [20]. All measurements were carried out in a texture analyzer (Lloyd Instruments™-Model TAPlus AMETEK, Elancourt, France), adjusting different probes for each test.

### 2.10. Content of Total Phenolic Compounds and Flavonoids

Extraction of phenolic compounds was performed following the methodology established by Alvarez-Parrilla et al. [14] with modifications. Flour and tortilla samples were dried, ground and stored as described for the extraction and quantification of carotenoids. Next, 5 g of the ground samples were sonicated for 30 min with 10 mL of methanol (JT Baker^®^, Fisher Scientific, West Palm Beach, FL, USA) (80%, *v*/*v*), centrifuged at 3500 rpm for 15 min and the supernatant was collected by filtration, adjusted to a volume of 25 mL, and kept in refrigeration at 4 °C for less than 12 h, until analysis. Total phenolic content (TPC) was determined by the Folin–Ciocalteu method and total flavonoids (TF) were determined by the AlCl_3_ method, according to the methodology described by de la Rosa et al. [21]. Results were expressed as milligrams of gallic acid equivalents (GAE) and milligrams of catechin equivalents (CE) per 100 g of sample (DW), respectively.

### 2.11. Antioxidant Capacity

The FRAP (ferric ion reducing antioxidant power), DPPH (2,2-diphenyl-1-picryl-hydrazyl-hydrate free radical) and ABTS (2,2’-azinobis-(3-ethylbenzothiazoline-6-sulfonate) assays were used to quantify the antioxidant capacity of the methanolic extracts of flour and tortillas samples, according to the methodology described by Alvarez-Parrilla et al. [14], de la Rosa et al. [21] and Brand-Williams et al. [22].

The extracts were obtained as described for the content of total phenolic compounds and flavonoids. For the FRAP assay, the FRAP reagent was prepared by mixing 0.3 M acetate buffer (Hycel^®^, Zapopan, Jalisco, México) with 10 mM TPTZ (2,4,6-tripyridyl-s-triazine; Acros Organics^®^, Morris Plains, NJ, USA) dissolved in 40 mM HCl (Hycel^®^, Zapopan, Jalisco, México), and 20 mM FeCl_3_ (Hycel^®^, Zapopan, Jalisco, México); in a ratio 10:1:1, v / v / v. The FRAP reagent was heated at 37 °C for 30 min and the assay was performed by mixing 180 µL of the FRAP reagent with 24 µL of sample in microplate wells. Absorption was measured at 595 nm every 60 s for 30 min in the UV–Vis microplate reader. The results were reported in millimole Trolox equivalent/g dry weight sample.

The DPPH assay was performed by mixing 50 µL of sample with 200 µL of the DPPH radical (190 µM in methanol; Merck^®^, Toluca, Estado de México, México) in microplate wells and absorbance was read at 515 nm for 10 min in the UV–Vis microplate reader.

For the ABTS assay, ABTS radical cation was prepared by diluting ABTS salt (7 mM; Merck^®^, Toluca, Estado de México, México) and K_2_S_2_O_8_ (2.45 mM; Merck^®^, Toluca, Estado de México, México) in phosphate buffered saline (PBS, pH 7.4, 0.15 M KCl; Merck^®^, Toluca, Estado de México, México), and the solution was incubated in refrigeration for 16 h. Then 12 µL of the sample was mixed with 285 µL of the ABTS radical cation in microplate wells and the absorbance was read at 734 nm for 30 min in the UV–Vis microplate reader. Results of DPPH and ABTS assays were reported as inhibition percentage.

### 2.12. Identification of Individual Phenolic Compounds in Ramón Seed Flour (RSF) by Ultra-Performance Liquid Chromatography Quadrupole Time of Flight Mass Spectrometry (UPLC-QTOF-MS)

Phenolic extracts of RSF, were cleaned up by solid phase extraction (SPE) using a C_18_ column (SupelCo, Merck^®^, Darmstadt, Germany) to reduce the presence of sugars and small organic acids. Briefly, 6 mL (1 volume) of extract was washed with 2 volumes of water (fractions discarded) and phenolic compounds were eluted with 2 volumes of pure methanol. Solvent was eliminated in a rotary evaporator and the dried extract re-dissolved in methanol high-performance liquid chromatography (HPLC) grade (2 mg/mL), passed through a nylon filter (0.45 μm) and applied into the chromatography system. Separation and identification of phenolic compounds was carried out by using an Infinity II LC System equipped with a photodiode array detector with a binary solvent pump and autosampler (Agilent Technologies, California, USA). Separation of individual phenolic compounds was carried out using a rapid-resolution high-definition (RRHD) reverse-phase C_18_ column (2.1 × 50 mm; 1.8 particle; ZORBAX Eclipse Plus^®^, Agilent, California, USA) at 25 °C, with a pre-column cartridge. The samples (1 μL) were injected and elution of phenolic compounds was completed in 12 min with a linear gradient and constant flow rate of 0.4 mL/min, as described before by Torres-Aguirre et al. [23]. The mobile phase consisted of solvent A (formic acid, 0.1% *v*/*v*, from Tedia^®^, Fairfield, Ohio, USA) and solvent B (acetonitrile from Tedia^®^, Fairfield, Ohio, USA). The linear gradients were as follows: 0–4 min, 90% A, 4–6 min, 70% A, 6–8 min, 62% A, 8–8.5 min, 40% A, 8.5–9.5 min, 90% A. Elution of phenolic compounds was detected at 255, 275 and 320 nm.

The LC equipment was coupled to a quadrupole time of flight (Q-TOF) mass spectrometer with electrospray ionization (ESI) source. The mass spectrometer was operated in negative mode and specific conditions as follows: capillary voltage of 4500 V, gas nebulizer pression 30 psi, dry gas (nitrogen) temperature of 340 °C and flow at 13 L/min. Mass range was monitored from 100 to 3000 m/z. Phenolic compounds were identified by comparing the accurate mass and isotopic distribution of their molecular ions [M − H]^−^, and in some cases their retention times with those of commercial standards, and compounds listed in a specialized database, using a find by database algorithm in the MassHunter Workstation qualitative analysis, version B. 07.00 (Agilent Technologies, Santa Clara, CA, USA).

### 2.13. Statistical Analysis

Data from microbiological analysis were compared with the permissible limits established by the Mexican regulation [9]. Physicochemical parameters, minerals, vitamins and phytochemical data were analyzed by Student *t*-test. Data from sensory descriptive analysis were analyzed using a repeated measure analysis of variance (ANOVA) and Fisher’s multiple comparisons (LSD) and data from acceptance test were analyzed Chi square test. All the analyses were carried out with XLSTAT program, version 2016.05 (Addinsoft^®^, Paris, France). The results are presented in mean values ± standard deviation (SD). The criterion for statistical significance was *p* < 0.05.

## 3. Results and Discussion

### 3.1. Tortilla Preparation and Food Safety

The preliminary sensory acceptance tests were considered for the selection of the RFT formulation, establishing the addition of 25% RSF for the tortilla. The lack of gluten in RSF influenced the properties of elasticity and firmness imparted by prolamines (gliadin) and glutelins (glutenin) [24]. Therefore, slight changes were made in the steps to prepare the tortilla (RSF), in order to obtain better physical characteristics. First, the kneading time was increased to 20 min and more water was added until a flexible and firm dough was obtained. In this way, higher hydration was achieved, breaking the endosperm protein bodies and promoting covalent and non-covalent interactions between larger polypeptides [25]. Second, water incorporation increased the elasticity and adhesiveness in the dough, so it was necessary to change the use of the rolling pin for a manual tortilla press, which is a simple, traditional and low-cost procedure. Third, salt content was increased by 25% in order to increase the hydration property of the proteins in RSF. Also, sodium facilitates denaturalization of proteins exposing their hydrophilic groups and increasing their degree of aggregation. This effect is generated by the increase of ionic force producing a reduction in protein solubility [26]. Finally, the cooking time was adjusted for RFT (26 s per side) according to the other changes made in the process, in order to avoid an impact on the consistency and rheology of the tortilla.

Microbiological analysis showed that both tortillas (RFT and WFT) were within the permissible limits, according to the Mexican legislation, in colony forming units of aerobic mesophilic bacteria (AC) (8 × 10^1^ and 1 × 10^1^ CFU/g, respectively. Permissible limit: 10,000 CFU/g), total coliforms bacteria (CC) (<1 × 10^1^ CFU/ g in each sample. Permissible limit: <30 CFU/g) and yeasts and molds (YM) (<10 × 10^1^ CFU /g. Permissible limit: 300 CFU/g) [11]. These microorganisms are indicators of management conditions or efficiency of the food preparation. They timely warn of inadequate handling or contamination that increases the risk for the presence of pathogenic microorganisms in the product [27].

### 3.2. Physicochemical and Micronutrient Characterization

Chemical and micronutrient composition of flours and tortillas is presented in Table 1. In flours, the contents of protein, ash, crude, and dietary fiber were higher in RSF than in commercial WF. In minerals, RSF was 2.5 times higher in copper (*p* = 0.01), 8 times higher in potassium (*p* < 0.01) and 2.3 times higher in sodium (*p* < 0.01) than WF. RSF was equal to WF in iron content (*p* = 0.13) and 6 times lower in zinc content (*p* < 0.01). RSF showed higher acidity and Vitamin C content (3.8 times) than WF, but carotenoid content was equal in both flours.

In tortilla, RFT retained more moisture (2.9%) than WFT, reflecting the extra water added in the wetting process, but the water activity was the same in both samples. Protein content was equal in RFT and WFT, showing RFT protein content was not affected by the partial substitution with RSF. RFT had an increase in the mineral content, showing an increase in copper (1.5 times higher) (*p* = 0.02) and potassium (1.8 times higher) in RFT (*p* < 0.01), while the content of iron (*p* = 0.84) and zinc (*p* = 0.81) remained similar. Dietary fiber was 4.5 times higher in RFT (14% DRV/100 g product) than in WFT (3% DRV/100 g product).

Little information has been published on the nutritional composition of the *Brosimum alicastrum* Sw. seed. A study carried out by Carter [8] showed that the dietary fiber content in ramón’s seeds from different countries (México, Honduras and Guatemala) was from 4.91 to 21.71 g/100 g (dry weight). In this study, RSF had a dietary fiber content of 13.0 g/100 g (fresh weight) or 14.9 g /100 g (dry weight), which is within the range reported by other authors [9,28]. Considering this fiber content, and according to the FDA criteria, RSF should be considered as food ingredient rich in dietary fiber (52.1% DRV/100 g) [29]. In comparing the mineral content of the flours, it is important to consider that commercial WF is enriched with folic acid, iron, and zinc, in accordance with Mexican regulations [11], which indicates that RSF is naturally rich in iron and would not need to be enriched to meet the legal iron requirements. This is particularly important for groups in poverty, for example iron deficiency is the main cause of anemia in Mexico, and is associated with a low intake of food from animal origin and a high intake of corn, with a high content of phytates that inhibit the iron absorption [30]. On the other hand, it is the first time that vitamin C or total carotenoid content of RSF has been reported. Both values were lower than those reported in lettuce using the same analytical technique [13], so we considered RSF was not a good source of these compounds.

Substitution of 25% WF by RSF had a positive impact in the mineral and dietary fiber content of RFT, so the new formulation showed a better nutrient profile and could have a positive impact on the consumer’s health. Minerals have important biological roles in the organism; for example, copper is an essential cofactor involved in the maturation of connective tissue, the synthesis of neurotransmitters and the prevention of cardiovascular diseases [31] while potassium participates in insulin secretion, creatine phosphorylation, carbohydrate metabolism, protein synthesis, nerve transmission and muscle contraction [32,33]. The potassium content in RFT was similar to that of bananas (358 mg/100 g) and higher than tomatoes (237 mg/100 g), which are considered as foods rich in this mineral [34]. The dietary fiber content in RFT (14% DRV/100 g) was higher than in corn tortilla (3.1% DRV/ 100 g product) [34], so RST can be considered as a good source of dietary fiber [29]. Guevara-Arauza et al. [35] reported a high fiber content in a tortilla added with nopal fiber (16.7%); nevertheless, this tortilla would provide 45% less protein than RFT. The importance of dietary fiber in food is well known; its consumption has shown benefits throughout the digestive process. Different physiological and prebiotic effects at the colon level make this nutrient a key component of a healthy diet [36]. Finally, the partial replacement of 25% WF with RSF did not provide a significant increase of Vitamin C or carotenoids in RFT.

### 3.3. Sensory Attributes and Consumer Acceptance

**Figure 1:**
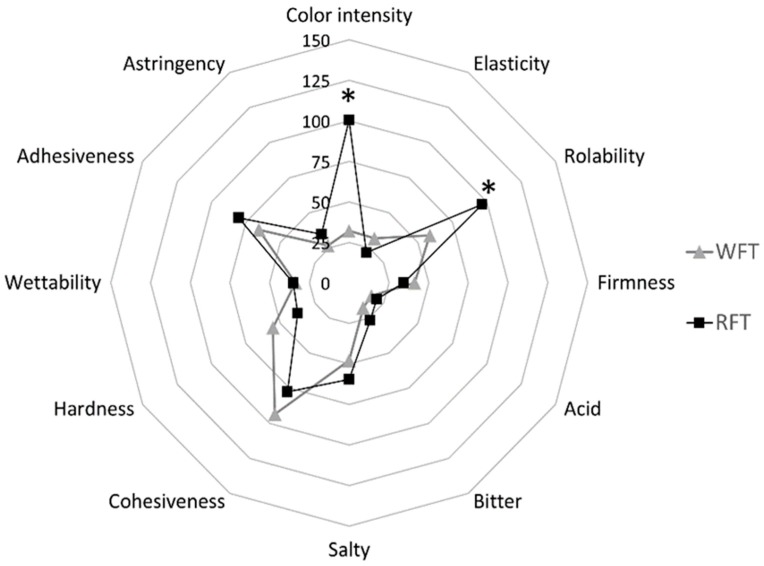
Sensory profile of tortillas. RFT—ramón flour tortilla, WFT—wheat flour tortilla. Intensity linear scale (150 mm). * Significant difference at *p* < 0.05.

The sensory characterization of both tortillas (RFT and WFT) in different attributes is shown in Figure 1. For a better interpretation of the intensity linear scale (150 mm), five intensity levels were considered: low (L, 0 to 37 mm), medium low (ML, 38 to 74 mm), medium (M, 75 mm), medium-high (MH, 76 to 112 mm) and high (H, 113 to 150 mm).

Of all the attributes evaluated in both tortillas, only two were perceived as significantly different between them: color and rollability. In color, RFT showed a MH intensity level (100.4 ± 9.4 mm), significantly different from WFT that was situated in L intensity level (32.2 ± 10.1 mm) (*p* < 0.01). Pantone^®^ scale was used to identify the color tones in both tortillas. RFT was located between Pantone codes # C 728C and 729C, indicating a light brown color, while WFT was classified with Pantone code # C 7401C, which corresponds to a light cream color. In tactile tests, RFT presented higher rollability (MH, 96.8 ± 23.1 mm) compared to WFT, which was in ML intensity (58.6 ± 24.8 mm) (*p* < 0.01). In the case of hardness, a trend was observed and WFT was perceived to be slightly harder (ML, 55.4 ± 24.0 mm) than RFT (L, 37.5 ± 14.7 mm) (*p* = 0.09). RSF color tones may be caused by the presence of tannins which are present in the maturation stages of certain seeds that change from green to brown due to morphological changes in the vascular tissue [37]; while WFT color tones are caused by Maillard reactions [38]. The increase of salt in RFT, added to promote hydration, was not detected in the salty taste. Cohesiveness and adhesiveness, which are directly related to the viscoelastic properties that gluten provides to create a support matrix capturing water and air molecules [39], were not affected by the water increase in RFT.

**Figure 2:**
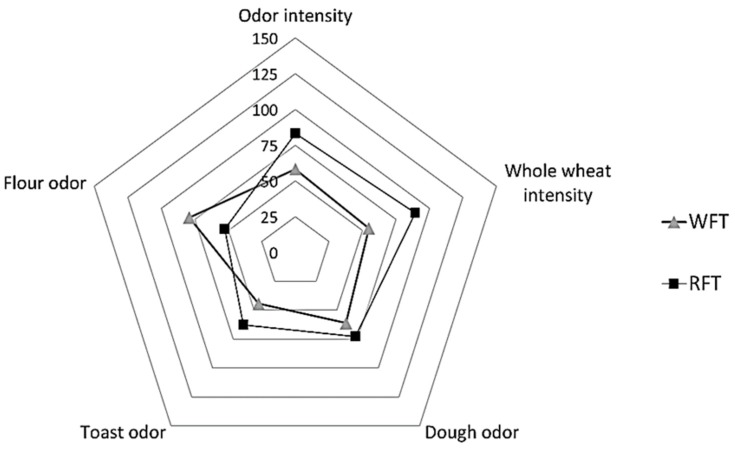
Sensory odor profile of tortillas. RFT—ramón flour tortilla, WFT—wheat flour tortilla. Intensity linear scale (150 mm).

In the olfactory phase, no significant differences were identified between tortillas, although RFT was perceived with a higher odor to whole wheat (MH, 90.0 ± 38.0 mm) (*p* = 0.06) and a less intense flour odor (ML, 52.8 ± 26.9 mm) (*p* = 0.07) in comparison to WFT (ML, 54.8 ± 25.9 mm and MH, 79.1 ± 27.0 mm, respectively). The smell of dough (RFT, 72.8 ± 34.5 mm and WFT 61.1 ± 36.4 mm) (*p* = 0.52) and toast (RFT, 62.8 ± 28.4 mm and WFT, 44.4 ± 27.5 mm) (*p* = 0.20) was found in a ML intensity in both tortillas (Figure 2).

**Figure 3:**
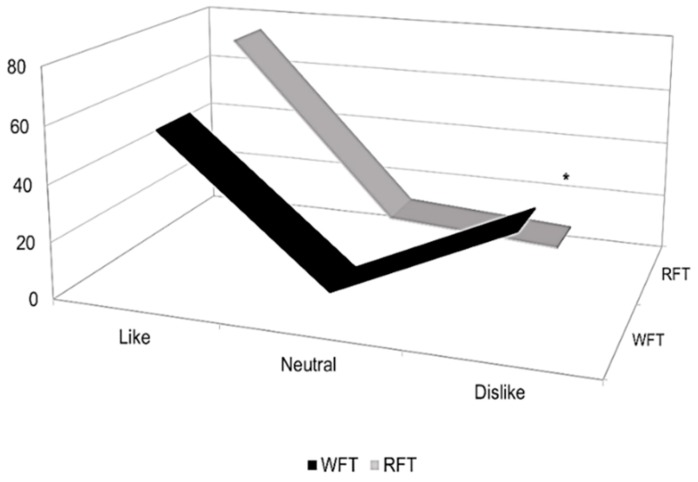
Consumer acceptance tortillas. RFT—ramón flour tortilla, WFT—wheat flour tortilla. * Significant difference at *p* < 0.05.

The final consumer acceptance was tested on 120 participants. The 9-point hedonic scale was divided into three areas (like, neutral and dislike). RFT and WFT were both liked by the consumer (*p* > 0.05) (Figure 3). However, 36% of consumers showed dislike for RFT and 10% for WFT (*p* < 0.01). RFT and WTF were placed in the following categories on the 9-point hedonic scale: “Like extremely” 2.5 and 4%, respectively (*p* = 0.72), “Like very much” 10.8 and 15%, (*p* = 0.44); “Like moderately” 22.5 and 40.8%, (*p* < 0.01); “Like slightly” 20.8 and 15.8%, (*p* = 0.40); “Neither like nor dislike” 7.5 and 14.2%, (*p* = 0.14) and “Dislike slightly” 20% and 5%, (*p* < 0.01). In general, 64% of consumers accepted RFT. Some consumer comments indicated that RST was milder, with balanced salt content, and whole wheat odor. These comments confirm the attributes evaluated by the judges, describing RST as softer (*p* = 0.06) and having a whole wheat odor too (*p* = 0.09).

RFT had a sensory acceptance similar to corn tortilla added with soy and amaranth (60%) [40] or corn tortilla added with *Brosimum alicastrum* [41]. Innovation in products with high cultural value could be an important factor for greater consumer acceptance. Studies in this regard are necessary in the development of functional foods appealing to different populations or ethnic groups.

### 3.4. Rheological Characterization

Data from the TPA test showed that there was a significant difference in gumminess (*p* < 0.01), adhesiveness (*p* = 0.01), elasticity (*p* = 0.01) and chewiness (*p* < 0.01) in the tortilla doughs (Table 2). RSF addition generated a less gummy, elastic and softer dough; requiring less force during mastication compared to the WF dough or other foods, such as white bread (10.8 Nm), lettuce (9.8 Nm) and carrot (12.7 Nm) [42]. It was also less adhesive than corn tortilla dough substituted with bean and amaranth flour (−0.36 N) and nopal flour and algae (−0.32 N) [43]. These results might be related to the extra water addition that participates in the dough structural changes and in the absorption capacity of flour starch granules, which affect the adhesiveness and hardness mass viscoelastic and tension properties [44]. In the same way, it has been reported that the starch granules of RSF are similar to the granules of potato, which possess superior gelling properties and better capacity of water retention than wheat starch [45]. 

The cohesiveness test was also performed on the tortilla dough, where RFT was slightly less cohesive than WFT dough. This indicates that the particles of the dough are less strongly bound in the dough with RSF [46] and therefore less effort is required to deform or break it. Therefore, it is considered that these behaviors could be produced by the primary structure of the RSF proteins, that could be low in thiol groups. In this way, there is a possibility that the lack of thiol–thiol reactions to form disulfide bridges, would make the dough collapse [47].

Cut test for the RFT required less strength and work than for the WFT (*p* < 0.01). Thereby, RSF provides more softness to the tortilla (Table 3), which means that it does not require a great effort to cut it with the incisors unlike other functional tortillas added with bean flour with amaranth (5.22 J) or nopal with algae (6.51 J) [43]. Another factor that could modify the rheology of the tortilla is the size of the starch granules of RSF (6.5 to 15 μm), which are smaller than those provided by wheat flour (11 to 41 μm), so it can be retro-degraded quicker in the cooking process [48,49]. In the multi-directional elongation and extensibility test, the same behavior was found as in the previous tests. The WFT was 10 times more resistant than RFT (*p* < 0.01) and with a similar elongation (*p* = 0.74), whereas extensibility in RFT required less force during extension (*p* < 0.01), it was less cohesive (*p* = 0.01) and it needed half the work to extend (*p* = 0.01). All these rheological characteristics are due to the interactions between globular proteins and starch molecules. The lower cohesiveness and other related rheological characteristics of RFT in comparison with WFT could be explained by considering that RSF proteins have a higher molecular weight, which would affect their extension properties generated by the release of CO_2_ during cooking and, hence, reduce the force of intermolecular interactions in the RFT [50,51]. A complete characterization of RSF proteins could help to better explain these behaviors and the rheological properties of RFT. Another factor that could be responsible for the lack of resistance in RFT may be the molecular rearrangement of polysaccharides with water during the cooking process, compromising the viscoelasticity and gelling of starch granules [52].

### 3.5. Polyphenolic Quantification and Antioxidant Capacity

RSF and RFT reported higher phenolic content than WF and WFT, respectively (*p* < 0.1). RSF showed higher total flavonoid content than WF (*p* < 0.01) and RFT and WFT were equal between them (*p* = 0.08) (Table 4). Compared with other studies, the total phenol content of RSF was higher than that reported by Tokpunar [53] for RSF (24.6 mg GAE/g), this could be due to genetic and environmental differences between *B. alicastrum* trees, which are wild trees with an extensive geographical distribution [9,24]. The phenolic content of RSF was also higher than that of other seeds, such as walnut (15.6–16.3 mg GAE/g sample), pecan nut (12.8–20.2 mg GAE/g sample), pistachio (8.7–16.6 mg GAE/g sample) or almond (2.4–4.2 mg GAE/g sample) [54] and 73 times higher than WF.

The content of phenolic compounds in RFT was 12 times higher than WFT and also exceeded the content of the previously mentioned nuts, and was similar to blackberry (27.1 mg GAE/g) [55]. In the same way, RFT was higher in flavonoids content than WFT, or other foods, such as blackberry (0.6 mg CE/g) [56], pistachio and almond (0.14 and 0.15 mg CE/g, respectively) [57]. In this sense, partial substitution of RSF in the tortilla samples provided a high phenolic and flavonoid contribution.

In relation to antioxidant capacity, RSF showed 48 times more activity than WF in the ABTS assay. Also, it was higher than blackberry (11.4 mmol TEAC/100g) [56] and equal to walnut (13.7 mmol TEAC/100 g) [58]. In the case of the tortillas, RFT reported twice as much antioxidant activity as WFT. The ABTS assay showed the highest values of antioxidant activity in all the samples. ABTS is sensitive to the presence of hydrophilic and lipophilic compounds, and a good correlation can be usually observed between the content of total phenols and antioxidant capacity [59], so it is a suitable technique to clarify the impact of RSF on the potential functional properties of RFT.

### 3.6. Identification of Individual Phenolic Compounds in RSF by UPLC-QTOF-MS

In addition to the nutritional value of RSF, the presence of a high content and diversity of phenolic compounds, many of which are strong free-radical scavengers, provides this product with added potential as a functional food. Twenty phenolic compounds were tentatively (comparison of high-resolution m/z value and isotope distribution) or positively (MS data plus retention time of available standards) identified in RSF (Table 5).

**Figure 4:**
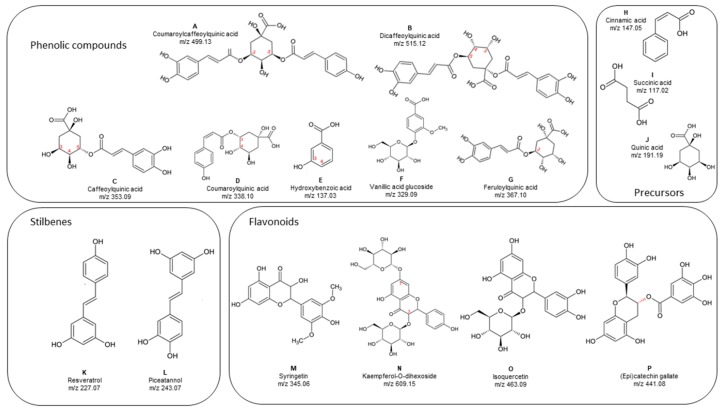
Structures of phenolic compounds tentatively or positively identified in RSF extract, by ultra-performance liquid chromatography quadrupole time of flight mass spectrometry (UPLC-QTOF-MS) analysis. Numbers in red indicate the possible isomers of each structure. Names of compounds (**A**–**P**) are provided in the figure and description of their identification by MS is given in the text.

Many of them are phenolic acids esterified with quinic acid. Compounds 3, 5 and 8 with Rt = 0.53, 0.94 and 1.54 min and a m/z = 353.0885, 353.0884, and 353.0882 were tentatively identified as isomers of caffeoylquinic acid, a derivate of caffeic acid esterified with quinic acid differing only in the position of esterification (C3, C4 or C5 of the aryl ring of quinic acid). Only compound **5** was positively identified as chlorogenic acid (Figure 4C). Compounds 16 and 17 were tentative identified as dicaffeoylquinic acid isomers with the same m/z = 515.1282 and 515.1206. These compounds have a structure of two moieties of caffeic acid and one of quinic acid linked through esterification at different positions into the aryl ring of quinic acid (Figure 4B). Compound 10 (Rt = 2.40 min, m/z = 367.1036) was tentatively identified as 3-O-feruloylquinic acid (Figure 4G). Compound **6** (m/z = 137.0244 and a Rt = 1.03 min) was tentatively identified as any of the possible isomers (*o*-,*m*-,*p*-) of hydroxybenzoic acid (Figure 4E). Compounds 9 and 12, wich eluted at Rt = 1.66 and 2.91 min respectively, with a m/z = 337.0936 and 337.0928, were tentatively identified as isomers of coumaroylquinic acid (Figure 4D). Compound 18 (Rt = 4.96 min, m/z = 499.1259) was tentatively identified as a coumaroyl-caffeoylquinic acid derivate (Figure 4A). Compound **1** (Rt = 0.47 and m/z = 329.0886) was tentatively identified as vanillic acid glucoside and was the only glycosylated phenolic acid (Figure 4F).

These compounds, linked by esterification with quinic acid, were the most numerous phenolic compounds in the RSF extract, representing half of the total compounds detected. Free quinic acid was also found (Compound 7, Figure 4J). This was expected since quinic acid is an organic acid abundant in plant tissues, that participates in metabolic routes as synthesis of lignin [60]. Succinic and cinnamic acids were also identified tentatively (Compounds 1 and 13, Figure 4I,H, respectively), both organic acids are precursors in the biosynthesis of flavonoids, isoflavones, and stilbenes [61].

Only a few flavonoids, stilbenes and their glycosylated derivates were identified in the RSF extract. Two stilbenes were tentatively identified, compound **20** (Rt = 7.06 and m/z = 227.0723) as cis or trans resveratrol (Figure 4K), and compound **19** (Rt = 5.58 and m/z = 243.0673) as piceatannol (Figure 4L), a more hydroxylated derivative of resveratrol. Compound 11 (Rt = 2.54 and m/z = 345.0628) was tentatively identified at as syringetin, a dimethylated flavonoid (Figure 4M). Compounds 14 and 15 (Rt = 3.73 and 3.86 with m/z = 609.1457 and 463.0894, respectively) were tentatively identified as glycosylated flavonoids: kaempferol 3-dihexoside (Figure 4N), whose structure contains two sugar moieties and isoquercetin, that is formed with quercetin and glucose (Figure 4O). Finally, one of the few free phenolics tentatively identified in RSF with Rt = 0.59 and m/z = 441.0813, was catechin gallate (Figure 4P). As described, the most abundant portion of phenolic compounds in the RSF extract consisted of phenolic acids esterified with quinic acid, while a minor portion was more heterogeneous containing flavonoids, stilbenes, and flavan-3-ols, mostly esterified with sugar moieties.

Only one study has been published identifying phenolic compounds in RSF, where hydroxycinnamic, gallic, vanillic, caffeic, and coumaric acids were identified in acid-hydrolyzed methanol extract and one flavonoid, epicatechin, was released by a continuous alkaline extraction [62]. In comparison with this previous study, a greater number of phenolic compounds were identified in RSF methanol extract in the present work. This was made possible by using the UPLC-QTOF-MS equipment whose main advantage is to provide a tentative identification of compounds for which no commercial standards are available, by using information about the molecular weight of individual phenolic compounds. To our knowledge, this is the first study to report a more detailed profile of phenolic compounds in ramón seeds using this technique, allowing for the identification of their native structures without alterations by hydrolytic reactions. This is important because acid and alkaline hydrolysis can degrade the glycosyl or ester linkages, but at the same time can fragment the structure of the phenolic compounds and alter their subsequent identification [63]. It is worth mentioning that no hydrolysable or condensed tannins were identified in RSF, which should be considered beneficial since tannins, despite their high antioxidant capacity are also known to interfere in nutrient absorption, which is not desirable in a product aimed at groups in poverty and/or malnutrition.

Derivatives of phenolic acids esterified with quinic acid, such as chlorogenic acid and the caffeoyl, coumaryl and feruloyl derivatives, found to be abundant in ramón seed, are typically found in coffee beans, which are recognized as a good source of healthy antioxidant compounds with health benefits like cardiovascular risk prevention. Special mention deserves those derivatives in which one quinic acid is esterified with two phenolic acid moieties; for example, dicaffeoil quinic acid, which has been found in medicinal plants and has a greater antioxidant activity than free phenolic acids [64]. The presence of stilbenes, such as resveratrol is also worth mentioning, although their abundance was low, and their identity should be confirmed. Resveratrol is a phenolic compound that demonstrates a wide range of health benefits as antioxidant, anti-inflammatory, and anti-proliferative in cancer cells. It has been identified in foods like peanuts, grapes and their products like roasted peanut butter and wine [65]. So, the fact that RSF can be considered a novel source of resveratrol for foods that regularly do not have it, such as tortilla or other food formulations, endows this seed with meaningful functional potential. The small number of flavonoids like syringetin, kaempferol and isoquercetin can further increase the healthy properties of RSF.

## 4. Conclusions

According to the results obtained in this study, *Brosimum alicastrum* Sw. seed flour (RSF) was a good source of protein, dietary fiber and minerals, such as copper and potassium, and a natural iron resource comparable to iron-fortified wheat flour (WF). In addition, RSF has a high antioxidant capacity (AC) and is rich in phenolic compounds, mainly chlorogenic acid and other phenolic acids esterified with quinic acid, although stilbenes and flavonoids were also present. The partial substitution of RSF (25%) in a wheat flour tortilla (RFT) increased dietary fiber, copper and potassium content. The sensory characteristics of RFT were like those of traditional flour tortilla (WFT), except for the light brown color and higher rollability. RFT was soft and less cohesive and it was accepted by 64% of consumers. Also, RFT increased 12 times its content of total polyphenolic compounds and twice its AC compared to WFT. Therefore, ramón seed flour improves the nutritional value of wheat flour tortilla and may provide potential functional properties that contribute to a healthier diet.

## 5. Patents

Patent request: MX/a/2018/011397. Dough of wheat flour and *Brosimum alicastrum* Sw. (ramón) flour for elaboration of food products, preferably tortilla.

## Figures and Tables

**Table 1:** Physicochemical characteristics of flour and tortillas samples.

Parameter	RSF	WF	RFT	WFT
Calories (Kcal)	336	349	350	357
Water (%)	13.3 ± 0.14 ^a^	12.9 ± 0.02 ^b^	25.0 ± 0.07 ^a^	22.1 ± 0.10 ^b^
Protein (%)	11.5 ± 0.39 ^a^	9.6 ± 0.16 ^b^	7.3 ± 0.11 ^a^	7.6 ± 0.10 ^a^
Fat (%)	0.6 ± 0.00 ^a^	0.6 ± 0.01 ^a^	9.6 ± 0.08 ^a^	8.9 ± 0.02 ^b^
Ashes (%)	3.4 ± 0.11 ^a^	0.6 ± 0.02 ^b^	3.4 ± 0.06 ^a^	3.1 ± 0.02 ^b^
Total Carbohydrates (%)	71.2 ± 0.56 ^b^	76.3 ± 0.17 ^a^	54.6 ± 0.16 ^b^	58.3 ± 0.15 ^a^
Crude Fiber (%)	3.4 ± 0.13 ^a^	0.4 ± 0.00 ^b^	0.9 ± 0.15 ^a^	0.2 ± 0.01 ^b^
Dietary Fiber (%)	13.0 ± 0.21 ^a^	1.6 ± 0.00 ^b^	3.6 ± 0.20 ^a^	0.8 ± 0.01 ^b^
pH	5.5 ± 0.01 ^a^	5.9 ± 0.01 ^b^	6.3 ± 0.01 ^b^	6.6 ± 0.02 ^a^
Activity water (Aw)	0.3 ± 0.02 ^a^	0.2 ± 0.01 ^b^	0.9 ± 0.02 ^a^	0.9 ± 0.01 ^a^
Titratable acidity (% CAE) *	0.004 ± 0.00 ^a^	0.001 ± 0.00 ^b^	0.002 ± 0.00 ^a^	0.001 ± 0.00 ^b^
Cupper (mg/100 g)	0.5 ± 0.10 ^a^	0.2 ± 0.00 ^b^	0.3 ± 0.00 ^a^	0.2 ± 0.00 ^b^
Potassium (mg/100 g)	1256.0 ± 12.00 ^a^	159.0 ± 5.00 ^b^	367.6 ± 13.00 ^a^	210.0 ± 10.00 ^b^
Iron (mg/100 g)	4.0 ± 0.70 ^a^	5.0 ± 0.20 ^a^	3.9 ± 0.20 ^a^	4.1 ± 1.20 ^a^
Zinc (mg/100 g)	1.0 ± 0.10 ^b^	6.0 ± 0.10 ^a^	4.5 ± 1.60 ^a^	4.9 ± 0.00 ^a^
Sodium (mg/100 g)	47.0 ± 0.10 ^a^	20.0 ± 0.10 ^b^	369.2 ± 0.40 ^b^	378.0 ± 0.60 ^a^
Vitamin C mg (Ascorbic acid/100g)	2.3 ± 0.01 ^a^	0.6 ± 0.06 ^b^	0.9 ± 0.09 ^a^	0.7 ± 0.03 ^a^
Carotenoids (mg β-carotenoids/100 g)	1.2 ± 0.10 ^a^	0.9 ± 0.10 ^a^	1.0 ± 0.00 ^a^	0.9 ± 0.00 ^a^

Mean ± SD. RSF—ramón seed flour, WF—wheat flour, RFT—ramón flour tortilla, WFT—wheat flour tortilla. * CAE—citric acid equivalent (0.064). Comparison between flours and between tortillas. Different letters indicate significant difference (*p* < 0.05).

**Table 2:** Rheological characteristics of tortillas dough samples.

Characteristic	RSF Dough	WF Dough
Gumminess (N)	10.2 ± 3.2 ^a^	39.1 ± 1.0 ^b^
Hardness (N)	34.1 ± 10.0 ^a^	60.5 ± 14.0 ^a^
Adhesiveness (J)	0.03 ± 0.0 ^a^	0.003 ± 0.0 ^b^
Elasticity (mm)	1.8 ± 0.0 ^a^	1.6 ± 0.1 ^b^
Chewiness (Nm)	1.9 ± 0.6 ^a^	6.3 ± 1.5 ^b^
Cohesiveness (Nm)	3.5 ± 0.1 ^a^	4.0 ± 0.1 ^b^

Mean ± standard deviation (SD). N—Newton, J—joul, mm—millimeter, Nm—newton for meter, RFS—ramón seed flour, WF—wheat flour RSF—ramón seed flour, WF—wheat flour. Comparison between doughs. Different letters indicate significant difference (*p* < 0.05).

**Table 3:** Rheological characteristics in tortilla samples.

Characteristic	RFT	WFT
Cutting force (N)	−0.8 ± 0.1 ^a^	2.6 ± 0.3 ^b^
Cutting work (J)	0.04 ± 0.0 ^a^	0.15 ± 0.0 ^b^
Elongation force (N)	0.3 ± 0.1 ^a^	3.1 ±0 0.0 ^b^
Elongation distance (mm)	3.9 ± 0.4 ^a^	4.1 ± 0.5 ^a^
Extensibility max force (N)	0.8 ± 0.0 ^a^	1.5 ± 0.2 ^b^
Rupture distance (mm)	3.3 ± 0.3 ^a^	3.2 ± 1.0 ^a^
Cohesiveness (N)	3.6 ± 0.2 ^a^	4.0 ± 0.0 ^b^
Work to max extension (J)	0.002 ± 0.0 ^a^	0.004 ± 0.0 ^b^

Mean ± SD. RFT—ramón flour tortilla, WFT—wheat flour tortilla. Comparison between tortillas. Different letters indicate significant difference (*p* < 0.05).

**Table 4:** Phytochemical content and antioxidant capacity of flour and tortillas samples.

			Antioxidant Capacity		
Samples	TPC	TF	DPPH	ABTS	FRAP
	mg GAE/g	mg CE/g	mmol TEAC/100 g	mmol TEAC/100 g	mmol TEAC/100 g
RSF	65.8 ± 2.26 ^a^	4.4 ± 0.18 ^a^	0.9 ± 0.09 ^a^	14.3 ± 0.10 ^a^	0.41 ± 0.04 ^a^
WF	0.9 ± 0.02 ^b^	0.1 ± 0.01 ^b^	0.0 ± 0.01 ^b^	0.3 ± 0.09 ^b^	0.04 ± 0.00 ^b^
RFT	21.1 ± 1.50 ^a^	0.7 ± 0.10 ^a^	0.3 ± 0.01 ^a^	0.4 ± 0.01 ^a^	0.04 ± 0.00 ^a^
WFT	1.8 ± 0.02 ^b^	0.5 ± 0.10 ^a^	0.2 ± 0.01 ^b^	0.2 ± 0.00 ^b^	0.04 ± 0.01 ^a^

Mean ± SD. All values are presented on dry weight basis. TPC—total phenolic compounds, TF—total flavonoids, DPPH -.2,2-diphenyl-1-picryl-hydrazyl-hydrate free radical assay, ABTS - 2,2’-azinobis-(3-ethylbenzothiazoline-6-sulfonate assay, FRAP -ferric ion reducing antioxidant power assay. GAE—gallic acid equivalents, CE—catechin equivalents, TEAC—trolox equivalents antioxidant capacity, RSF—ramón seed flour, WF—wheat flour, RFT—ramón flour tortilla, WFT—wheat flour tortilla. Comparison between flours and between tortillas. Different letters indicate significant difference (*p* < 0.05).

**Table 5:** Retention times, and characteristic ions of phenolic compounds found in RSF extracts.

Compound	Tentative Identification	Formula	Rt (min)	m/z [M − H]^−^	Measured Mass	Exact Mass	Δm ppm	Abundance
1	Vanillic acid glucoside	C_14_H_18_O_9_	0.47	329.0886	330.0961	330.0951	3.02	5170
2	Succinic acid	C_4_H_6_O_4_	0.47	117.0195	118.0265	118.0266	−0.84	2542
3	Caffeoylquinic acid	C_16_H_18_O_9_	0.53	353.0885	354.0960	354.0951	2.54	86,163
4	Catechin gallate	C_22_H_18_O_10_	0.59	441.0813	442.0886	442.0902	−3.16	1111
5	Chlorogenic acid *	C_16_H_18_O_9_	0.94	353.0884	354.0958	354.0951	1.97	122,874
6	*m*−Hydroxybenzoic acid	C_7_H_6_O_3_	1.03	137.0244	138.0318	138.0317	0.72	11,067
7	Quinic acid	C_7_H_12_O_6_	1.52	191.0563	192.0632	192.0634	−1.04	13,344
8	Caffeoylquinic acid	C_16_H_18_O_9_	1.54	353.0882	354.0956	354.0951	1.41	51,125
9	p−coumaroylquinic acid	C_16_H_18_O_8_	1.66	337.0936	338.1006	338.1002	1.18	8509
10	3−O−Feruloyl quinic acid	C_17_H_20_O_9_	2.40	367.1036	368.1109	368.1107	0.54	17,141
11	Syringetin	C_17_H_14_O_8_	2.54	345.0628	346.0703	346.0689	4.04	5800
12	p−coumaroylquinic acid	C_16_H_18_O_8_	2.91	337.0928	338.1001	338.1002	−0.29	8910
13	Cinnamic acid	C_9_H_8_O_2_	2.97	147.0448	148.0519	148.0524	−3.37	4009
14	Kaempferol−O−dihexoside	C_27_H_30_O_16_	3.73	609.1457	610.1544	610.1534	1.63	3113
15	Isoquercetin	C_21_H_20_O_12_	3.86	463.0894	464.0968	464.0955	2.80	2519
16	Dicaffeoylquinic acid	C_25_H_24_O_12_	4.16	515.1204	516.1282	516.1268	2.71	51,207
17	Dicaffeoylquinic acid	C_25_H_24_O_12_	4.29	515.1206	516.1276	516.1268	1.55	82,208
18	*p*−Coumaroyl−caffeoylquinic acid	C_25_H_24_O_11_	4.96	499.1259	500.1329	500.1319	1.99	3648
19	Piceatannol	C_14_H_12_O_4_	5.58	243.0673	244.0748	244.0736	1.63	1112
20	Resveratrol	C_14_H_12_O_3_	7.06	227.0723	228.0784	228.0786	−0.87	1669

Abbreviations: Rt. Retention time * Identification confirmed by commercial standards.
